# Vivisection: The Anti-Vivisection Society

**Published:** 1900-01

**Authors:** 


### TEXAS MEDICAL JOURNAL.

AUSTIN, TEXAS.

A MONTHLY JOURNAL OF MEDICINE AND SURGERY.

F. E. DANIEL, M. D., Editor.
S. E. HUDSON, M. D., Managing Editor.

Published Monthly at Austin, Texas, by Drs. Daniel and Hudson. Subscription price $1.00 a year in advance.

Eastern Representative: John Guy Monihan, St. Paul Building, 220 Broadway, New York City.

Official organ of the West Texas Medical Association, the Houston District Medical Association, the Austin District Medical Society, the Brazos Valley Medical Association, the Galveston County Medical Society, and several others.

### Vivisection: The Anti-Vivisection Society.

That readers may understand what's up, and what kind of an enemy the medical profession have to reckon with, and must get a move on them, we reproduce in "Abstracts and Selections," and call attention to, a letter from the President of the American Medical Association, in the editorial columns of the *Journal* of the Association. The Anti-vivisection bill is up in Congress again.

**FOOLS OR KNAVES?**

As to the "Society."—We have received several copies of a little pamphlet called "The New England Anti-Vivisection Society Monthly." It is published anonymously at Boston, and no names are given of the officers or of those who compose the "society." We see nothing said about "bond" of officers; there is no responsible head,—so far as is shown,—no trustees,—no one to account for, or account to for the large amounts of money that will doubtless be paid in by cranks, degenerates, gullibles and hysterical people of both sexes ostensibly in aid of "prevention of cruelty to animals." Yet we observe that "life membership is $100," and the promoters say they expect to get several thousand members. That is,—several hundred thousand dollars. What assurance will the "life members" or any other contributors have that the money will be expended,—

in the crusade of ignorance and malice which these persons announce it is the purpose to prosecute? Aye; there's the bug under the chip. Greed is the spur that pricks the side of their intent. They are playing upon the sympathies of the ignorant and credulous, and have a soft snap.

That readers may see what methods these concealed individuals are pursuing to feather their nests, we reproduce the platform of the "society" from the front page of their "Monthly"; it is kept standing. Here it is:

"WHAT IS VIVISECTION?

"Answer. Vivisection is the mutilating, cutting and burning of living animals; they are dissected, roasted, boiled and skinned, when alive and in full possession of all their faculties; nerves are dissected out, laid bare and connected with the poles of a powerful electric battery from which currents of electricity are passed over these nerves; this probably causes the greatest agony of which sentient beings are capable. At the present time the only way to attack this horrible crime is by printing and circulating information so as to let the public know just what vivisection is, and how much it is done.

" 'The New England Anti-Vivisection Society' has been organized and incorporated for this purpose. In order to accomplish its object it must have money to pay its office rent, and for printing and postage; every additional member increases its influence, apart from the income from membership; at present it has several hundred members, and it wants to have several thousand before the end of another month; every dollar given aids the work directly; all of its officers serve without pay, a thing that can be said of no other society of its kind in this State; annual membership is five dollars; associate membership is one dollar, which does not give the right to vote; life membership is one hundred dollars.

" 'The New England Anti-Vivisection Society' opposes vivisection

"First.—Because the number of animals vivisected, with unknown and inconceivable agony to each one, is probably several thousand each day.

"Second.—Because anæsthetics are very seldom efficiently used.

"Third.—Because the results of vivisection are as near to absolute worthlessness as it is easv to get—in fact, cause great and absolute harm.

"Fourth.—Because, as recent revelations in the *Transcript* and other papers abundantly show, vivisectors of prominence and supposed character are, almost without exception, untruthful, and cannot be relied on to speak the truth about their acts.

"Fifth.—Because the animals used for vivisection are largely pet dogs and cats which are stolen for the purpose. The *Evening Record* has exposed one of the greatest medical colleges in Massachusetts in its attempt to seduce a supposed characterless wretch to steal cats for vivisectionists (which he freely admitted he intended to do), without any expressed disapproval on the part of said college.

"Sixth.—Because the practice of vivisection has bred and is breeding, in vivisectors, a degree of cowardice, meanness, hypocrisy and insane lust for cruelty, that, it is to be feared, will produce, in another generation, a race of educated monsters of depravity such as this world has not known and the imagination of mankind cannot conceive. Already human victims are openly demanded by vivisectors in Ohio, and every true vivisector longs, in his heart, for HUMAN VICTIMS to vivisect.

"Just as soon as encouragement and the present tacit acquiescence in vivisection has changed the vivisector from the cowardly hypocrite he at present is, into the arrogant persecutor he longs to be, the demand for men and women will be universal—not confined to a single State!

"Please join the 'New England Anti-Vivisection Society' *at once,* and further aid this best of all causes by inducing your friends to join. Also, help circulate the Society's literature.

"N. B.—The above statements are not merely speculative, but are vouched for by countless well-known scientists, surgeons and physicians of the highest possible character."—*From N. E. Anti-Vivisection Monthly.*

I THINK I never saw more ignorance, malice, misrepresentation, perversion of fact,—more downright mendacity crowded in so small a space. Voltaire says: "Some people have more zeal than understanding." These people either have "understanding," and know that what they state is false, in which case they are knaves, or they are ignorant of what they pretend to "expose"—in which case they are over zealous fools. I incline to the first proposition. I think they know better,—have "understanding" all right, but are playing upon the weak and credulous for profit. Now, as to Senator Gallinger, who is engineering the bill in Congress,—their tool, evidently, he probably does not know any better; he's a homeopath, and before he got to be senator was practicing homeopathy,—alleged—somewhere. Homeopaths have no need to know anything of physiology or pathology, as they ignore both, and "practice" by a little book of "symptoms" and "provings." It is hardly to be supposed that he knows anything of the history of vivisection, and the rise of physiology; I doubt if he ever heard of Wharton Jones, Huxley, Brown-Sequard, Bernard, Webber, Waller, the immortal Ludwig, or even of Flint, Dalton or Welch. My private opinion is that these fellows are "working" him. Whether he will come in on the "divvy," and share that prospective hundred thousand dollars is a matter we cannot speculate on; that's further along.

I once heard a fellow say that his mother advised him to "never fight a fool or argue with an idiot." We have to do both in a case like this; and however we may denounce their methods, or impugn their motives, they will get in their work on Congress if those in the profession who have political pulls are not up and doing. Should such bill be passed it would be an everlasting disgrace to America. Yet, Congress is liable to do any fool thing; Congress once paid $33,000 for an alleged "cure for hydrophobia," which, upon analysis, was found to be composed in part, not of the hair of the dog, but of the excrement.

**AS TO VIVISECTION.**

It would seem useless to contradict the absurd statements made in the pronunciamento of these people published above; they refute themselves. For instance, that vivisection is done without anæsthetics; that the results of vivisection are absolutely worthless; that the great investigators,—the immortals who, by vivisection, founded the science of physiology, and enriched medical science by their discoveries in biology, are liars; that it is proposed to dissect the living human animal, etc. Every reader of medical literature knows that they are false. Regarding the use of anæsthetics, were one a brute, as said by these cranks, and incapable of pity and devoid of all sense of humanity, he would use an anæsthetic to paralyze motion and facilitate dissection. I feel that it is hardly worth while to treat such statements seriously, but in case there should be any who read this who do not know what vivisection has done for science, I will cite just three discoveries, which may be said to have laid the foundation of modern physiology, revolutionized our ideas as to nutrition, the functions of the nervous system, and the mechanism of the circulatory system, and gave us all the knowledge we have of these subjects:

1. The Webber brothers, by vivisection (1845), ascertained that "an impulse sent down the vagus nerves will stop the heart from beating; while impulses reaching the heart along the cardiac sympathetic nerves from the thoracic spinal cord stir up the heart to more vigorous or frequent beats." Hence our first conceptions of "inhibition."

2. Claude Bernard's discovery of the formation of glycogen in the liver (1851) "came," says Prof. Michael Foster,* "like a bolt from the blue," and he adds: "It was the first realistic proof of the synthetic powers of the animal organism." * * * "The knowledge that the same hepatic cell was engaged both in secreting bile

---

*See closing paragraph of this article.—Ed.

and manufacturing glycogen, and that the sugar or other products of digestion were carried from the intestine, not straight to the tissues which they were destined to nourish, but to the liver, there to undergo transformation and await some future fate, marked the beginning of a new way of looking at the problems of nutrition."

3. Claude Bernard (1851) divided the cervical sympathetic (in a turtle) and it "led to a widening of the blood-vessels and a warming of the ear and other parts of the head and neck." This was the beginning of the great vaso-motor knowledge. Waller and Brown-Sequard had previously shown that stimulation of the peripheral part of the divided sympathetic constricted the blood-vessels and reduced the temperature, says Prof. Foster, but "Bernard's experiment marks the beginning of our vaso-motor knowledge." Thus was a knowledge of the mechanism revealed, whereby the distribution of blood supply is regulated,—and an explanation afforded why a man may not bleed to death in his own veins.

It would be a waste of time and energy,—and I have neither to spare,—if not supererrogation, to point out to any educated reader the bearings of these important discoveries on medical science and medical practice. For fuller accounts of the labors and discoveries of the Webbers, Bernard, the immortal Ludwig, and others, see *Smithsonian Reports* for 1896, page 350 *et seq.*, article "Recent Advances in Science," being the introductory to The Huxley Lectures, by Prof. Michael Foster, Sec. Royal Society, England; and same volume, page 365, article "Ludwig and Modern Physiology," by J. Burdon Sanderson, a lecture delivered at the Royal Institute, January 24, 1896; reproduced from *Science Progress,* Vol. V, pages 1-21, 1896.

The above should be sufficient to show how "absolutely worthless" are "the results of vivisection," as alleged by these pseudo humanitarians.

The Western Texas Medical Association, in session at San Antonio, have taken action in the important matter discussed in the above, and we urge all other Texas medical societies and medical men to act at once.

